# Convex Decomposition for a Coverage Path Planning for Autonomous Vehicles: Interior Extension of Edges

**DOI:** 10.3390/s19194165

**Published:** 2019-09-25

**Authors:** Lasse Damtoft Nielsen, Inkyung Sung, Peter Nielsen

**Affiliations:** 1 Department of Mathematical Sciences, Aalborg University, 9220 Aalborg, Denmark; 2 Operations Research Group, Department of Materials and Production, Aalborg University, 9220 Aalborg, Denmark

**Keywords:** convex decomposition, cellular decomposition, edge extension, coverage path planning, aerial surveillance and monitoring

## Abstract

To cover an area of interest by an autonomous vehicle, such as an Unmanned Aerial Vehicle (UAV), planning a coverage path which guides the unit to cover the area is an essential process. However, coverage path planning is often problematic, especially when the boundary of the area is complicated and the area contains several obstacles. A common solution for this situation is to decompose the area into disjoint convex sub-polygons and to obtain coverage paths for each sub-polygon using a simple back-and-forth pattern. Aligned with the solution approach, we propose a new convex decomposition method which is simple and applicable to any shape of target area. The proposed method is designed based on the idea that, given an area of interest represented as a polygon, a convex decomposition of the polygon mainly occurs at the points where an interior angle between two edges of the polygon is greater than 180 degrees. The performance of the proposed method is demonstrated by comparison with existing convex decomposition methods using illustrative examples.

## 1. Coverage Path Planning

Coverage path planning (CPP) is the problem of determining a path that guides a vehicle or a machine to cover an area of interest, while avoiding obstacles inside of the area [1]. CPP has become significant with the increase of use of autonomous vehicles in several applications [2,3,4]. In particular, recent advances in the research of Unmanned Aerial Vehicles (UAVs) and their applications to surveillance and monitoring of an area have made the problem much more popular [5,6,7].

**Figure 1:**
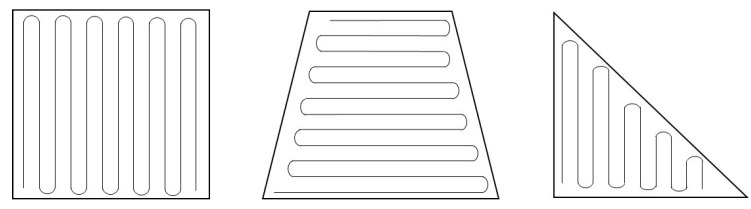
Examples of coverage paths on convex polygons.

When the area to cover is simple and convex (a polygon is a convex polygon when the interior angles of all points of a polygon are less than or equal to 180 degrees), CPP is rather easy to achieve. Multiple lines parallel to one of boundaries of a polygon can be a solution to the CPP. Based on this idea, the boustrophedon (meaning the way an ox walks from Greek, which specifically means the area is covered in parallel lines [8,9]) method is commonly applied to obtain the path, as illustrated in Figure 1.

**Figure 2:**
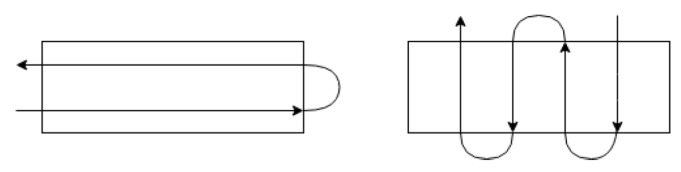
The number of turns, depending on the direction of the lanes.

In general, the boustrophedon method is applied to the CPP such that the number of turns in a path generated is minimized, because turning a vehicle often requires more energy and time than a straight movement. Following this fact, the path on the left in Figure 2 is more preferable than the path on the right.

Formally, with the definition of the width of a convex polygon as follows, the number of turns of a coverage path for a convex polygon is minimized when the path is generated perpendicularly to the direction of the width of the polygon.

**Definition** **1.** *The altitude, A, in any direction, for a given convex polygon P, is the greatest perpendicular distance between two lines that intersect P and which are parallel to the direction.*

**Definition** **2.** *The width W, for a given convex polygon P, is the minimum altitude.*

**Figure 3:**
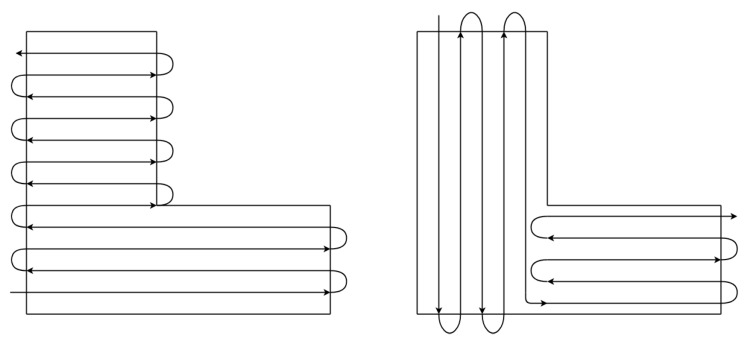
Coverage paths in a concave polygon.

As the area becomes complex and concave, however, it is difficult to apply the boustrophedon method to the CPP. As illustrated in Figure 3, for a concave polygon, a coverage path with a single direction (left) would be worse than a coverage path with multiple directions, (right) in terms of the number of turns.

**Figure 4:**
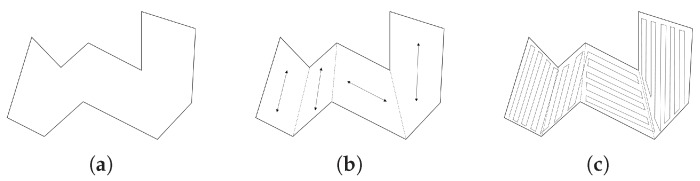
The concept of the coverage path planning (CPP) solution approach: (**a**) An area of interest; (**b**) a convex decomposition solution with covering directions; and (**c**) a complete coverage path, found by connecting sub-paths of sub-polygons.

One solution approach handling the concavity of an area is to decompose the area into convex sub-areas. This approach is referred to as a cellular decomposition, which breaks an area of interest into simple and non-overlapping regions called cells [1]. Aligned with the CPP in a convex polygon, the convex decomposition is often carried out such that the sum of widths of the decomposed convex sub-polygons is minimized. After the decomposition, the boustrophedon method can be applied to each sub-polygon to obtain coverage paths, and a complete coverage path for the original area is finally generated by connecting the sub-coverage paths for sub-polygons. The path connection is often formulated as a Traveling Salesman Problem (TSP) which minimizes the travel distance/time to visit all decomposed sub-polygons. The concept of the CPP solution approach is described in Figure 4.

**Figure 5:**
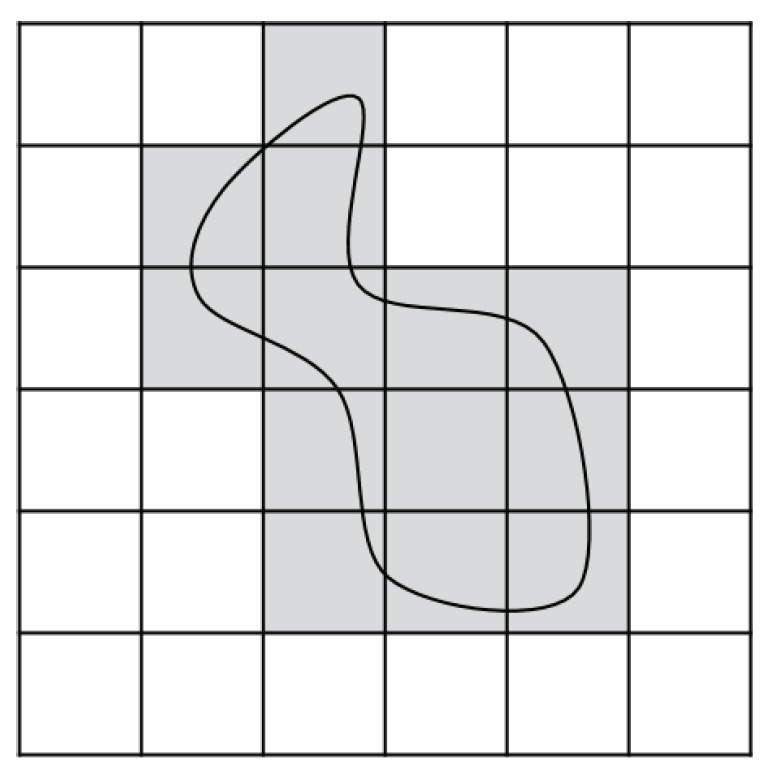
An example of a grid map.

Note that the grid-based approach, which divides an area of interest into a collection of uniform grid cells, has also been applied to solve the CPP [1]. A grid represents a free space to move or an obstacle. Then, a sequence of grids, derived following a certain logic, corresponds to a coverage path. Figure 5 shows an example of a grid map with an obstacle inside. Grids with an obstacle are shaded.

Obviously, the quality of a coverage path derived by the grid-based approach (i.e., the completeness of the path to cover a target area) depends on the resolution of the grid. As a high-resolution grip map for a high-quality coverage path exponentially increases the complexity of a path finding algorithm and memory usage, grid-based coverage methods are suitable only for relatively small-sized areas (e.g., from indoor mobile operation) [1], which highlights the applicability of the CPP with the convex decomposition.

Lastly, it should be noted that convex decomposition is also integral to multi-unit CPP scenarios, where segmentation of an operation area is important for efficient use and security of the resources. In a searching and mapping scenario using multiple UAVs, the area of interest should be segmented into reasonable subsets, which can be shared among the UAVs while ensuring that the subsets are covered safely with minimum effort.

As such, convex decomposition plays a key role in coverage operations (i.e., surveillance and monitoring) using unmanned platforms, under both single- and multiple-vehicle scenarios. The decomposition is critical to minimize the effort to cover a region (typically found by reducing the number of turns) and assign tasks to vehicles in a proper manner. In this paper, considering their importance, we review existing convex decomposition methods and propose a new convex decomposition method which is simple but well-performing.

## 2. Existing Convex Decomposition Approaches

To decompose concave polygons into convex sub-polygons, several approaches have been proposed. A common approach is based on a sweep line method often called as trapezoidal decomposition [10]. This method uses a sweep line in a direction of a polygon and splits the polygon parallel to the sweep direction whenever the line intersects a vertex of the polygon. This is fast, but leads to redundancies in the decomposition by nature, resulting in more sub-polygons than necessary.

**Figure 6:**
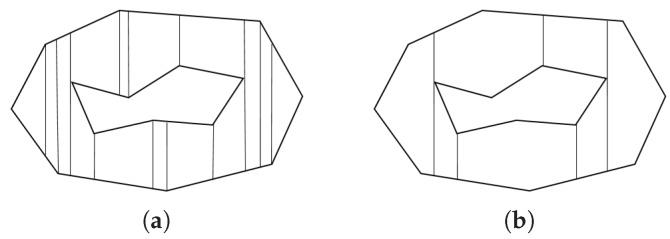
Comparison between (**a**) the trapezoidal decomposition and (**b**) the method proposed in [8].

Inspired by this drawback, [8] proposed an advanced sweep line algorithm, which handles the redundancies in the trapezoidal method by merging sub-polygons according to certain conditions. In the work of [8], sweep line decomposition was conducted multiple times for each sweep direction perpendicular to each boundary of a target polygon, in order to find the best sweep direction. Figure 6 describes the differences between outputs from the trapezoidal decomposition and the sweep line method proposed in [8], with the sweeping direction from left to right.

Instead of having a single sweep direction, [11] ran the sweep line method multiple times independently with multiple sweep directions and overlaid all decompositions obtained, splitting a target polygon into many sub-polygons. From the initial decomposition, [11] used dynamic programming to merge the initial sub-polygons into final sub-polygons, with respect to minimizing the sum of widths of the final sub-polygons. While the method in [11] demonstrated better performance than that of [8], the method has exponential time complexity, as it searches for as many combinations of initial sub-polygons as possible.

However, sweep line-based convex decomposition has a clear limitation. The decomposition cannot handle more than one vertex intersecting a sweep line. Thus, given a sweep line and its direction, all vertices in a polygon need to be unique with respect to the sweep line, implying a poor performance for concave polygons with rectilinear shapes. Furthermore, it is difficult to determine an optimal sweep direction as a target polygon becomes complicated.

Morse-based cellular decomposition [12,13] is a more general decomposition method (of which the sweep line method is a special case) which can handle non-polygon shapes. It is able to decompose an area into sub-areas defined by non-linear tracks (e.g., circles or spirals).

Heuristic approaches for convex decomposition have also been proposed. The methods presented in [5,14] minimized the number of turns using a recursive greedy method which splits a polygon at concave vertices. The proposed algorithm has proved to have polynomial time complexity. However, as it is a greedy algorithm, its performance depends on the problem instance [8].

As reviewed, the existing convex decomposition approaches have drawbacks, in terms of their performance in minimizing the sum of widths of convex sub-polygons or computational complexity to obtain a solution. In this paper, motivated by the room left to further improve such convex decomposition methods, we propose a new convex decomposition method with a focus on the following aspects:

- Simple and fast to find a decomposition solution;
- applicable to any shape of polygon (in particular, to rectilinear shapes); and
- provides a competitive decomposition solution, compared with existing methods.

The remainder of this paper is structured as follows: In Section 3, we describe the proposed method with an example to clarify the method. In Section 4, we analyze the method by comparing it with the existing convex decomposition methods. The extension of the proposed method to multi-unit CPP is discussed in Section 5. Finally, we conclude our study with remarks on future studies in Section 6.

## 3. Proposed Solution

### 3.1. Interior Extension of Edges

The proposed algorithm is designed based on the idea that the convex decomposition mainly occurs at the points where the interior angle (the angle towards the area to be covered) between two edges of a target polygon is over 180 degrees.

**Figure 7:**
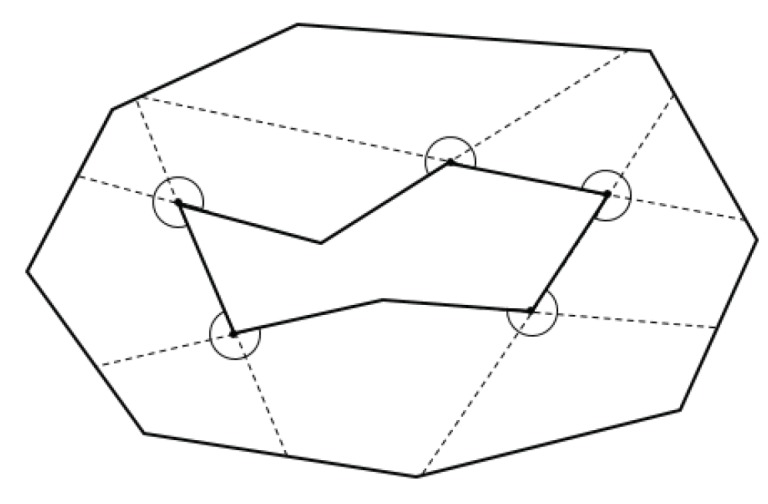
Interior extension of edges.

Following this idea, we first extend the edges of a polygon that form an interior angle greater than 180 degrees, until they hit the perimeter of the target polygon or an obstacle inside the polygon. In doing so, all the sub-polygons generated by the edge extension are convex. Figure 7 illustrates this concept, using the example of Figure 6.

In Figure 7, there are five points, where their interior angles are greater than 180 degrees. The ten corresponding edges are extended until they hit the boundaries of the target area, resulting in ten initial convex sub-polygons. The extended edges are denoted as dotted lines in the figure.

### 3.2. Merging Initial Sub-Polygons

These initial sub-polygons found by edge extension can be further merged with adjacent ones, as long as the merged polygons are convex. Importantly, this polygon merge reduces the number of the final sub-polygons and the total sum of widths of the sub-polygons, which is the main objective of convex decomposition.

**Figure 8:**
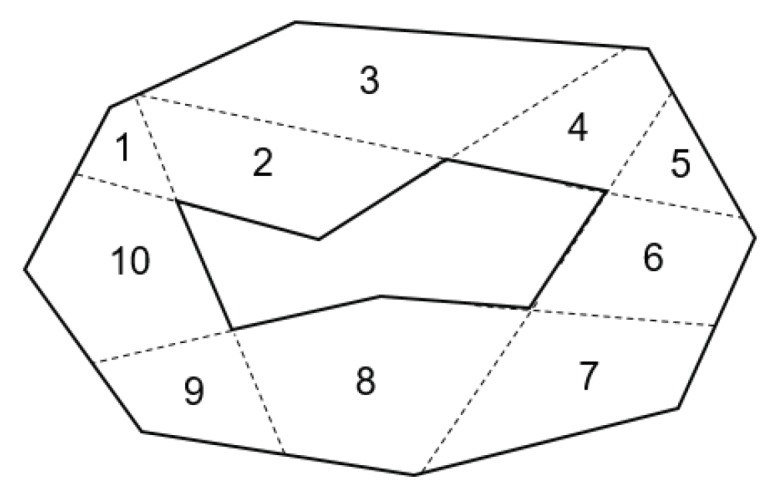
Initial sub-polygon set with identifiers.

Given a set of initial sub-polygons found by edge extension, we first find all combinations of the initial sub-polygons which can be merged as convex polygons, termed as convex merge options. For example, given the ten initial sub-polygons with index numbers as presented in Figure 8, there are 25 convex merge options of the initial sub-polygons: Ten cases for a single sub-polygon, ten cases for two initial sub-polygons, and five cases for three initial sub-polygons (i.e., {(1),⋯,(10),(1,2),⋯,(1,10),(1,2,3),⋯,(1,9,10)}).

After computing all convex merge options, we then select specific options such that the sub-polygons generated by the selected options cover the original area of interest and the total width of the sub-polygons is minimized. To cover the original area, all initial sub-polygons must be included in one of the convex merge options selected.

This convex merge option selection is a set partitioning problem. Formally, let *S* denote the set of initial sub-polygons indexed by *i*. The set of convex merge options, denoted by Ω, is indexed by *j*. The coefficient $w_{j}$ is the width of the convex polygon generated by convex merge option j∈Ω (refer to [8,15] for the details about algorithms for finding the width of a convex polygon). With the integer decision variable $\lambda_{j}$, which equals one if merge option *j* is selected, and zero otherwise, the convex merge option selection can be formulated as an Integer Programming (IP) model, written as follows:

(1) $$\begin{array}{cc} \; & \min\;\sum_{j\in \Omega }w_{j}\cdot \lambda_{j}, \end{array}$$

(2) $$\begin{array}{ccc} s.t.\; & \sum_{j\in \Omega }a_{ij}\cdot \lambda_{j}=1 & \forall i\in S, \end{array}$$

(3) $$\begin{array}{ccc} \; & \lambda_{j}\in \left\{0,1\right\} & \forall j\in \Omega , \end{array}$$

where $a_{ij}$ is one if the merge option *j* includes the initial sub-polygon *i*, and zero otherwise. The objective function (1) minimizes the total width of the sub-polygons. The constraint (2) ensures that all initial sub-polygons are included in one of the convex merge options selected. The constraint (3) is an integrality constraint on the decision variable.

**Figure 9:**
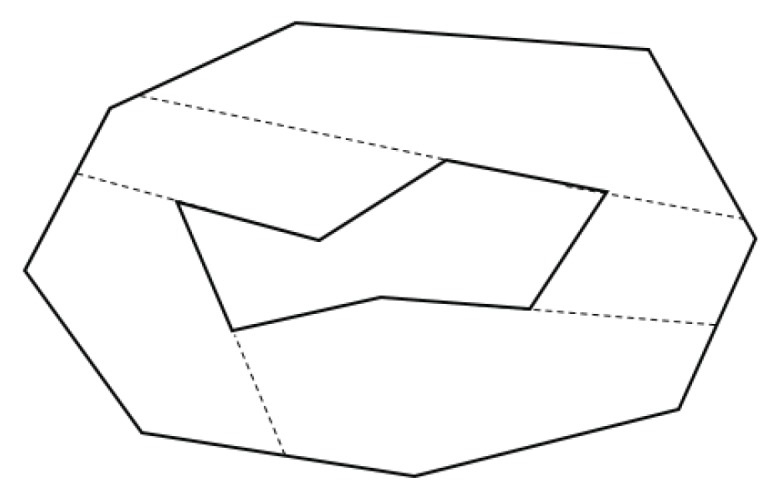
Final solution of the proposed method.

For the illustrative example in this section, the optimal solution of the IP model is presented in Figure 9, where five convex merge options {(1,2),(3,4,5),(6),(7,8),(9,10)} are chosen.

### 3.3. Implementation

The first step of the convex merge option selection, which seeks all possibilities for merging the initial sub-polygons as convex polygons, takes a long time as the number of the initial sub-polygons increases. Given *N* initial sub-polygons, $\sum_{i=1}^{N}{}_{N}C_{i}$ combinations should ideally be checked, in order to find whether the combinations return convex polygons or not, while computing their widths. From our initial test, we observed that it became difficult to complete this step quickly when the number of the initial polygons was great (e.g., N=300).

To overcome this difficulty, we implemented an approximation scheme to limit the search for convex merge options. Suppose that an initial sub-polygon set *S* is generated after the edge extension is given (see Figure 8). All convex merge options using the initial sub-polygons are then found. For this step, we sequentially search convex merge options that consist of *n* of the initial sub-polygons, in ascending order of *n* (i.e., from one to |S|).

It should be noted that, as an approximation, convex merge options consisting of *n* initial sub-polygons are only found by checking possible combinations of convex merge options consisting of ⌊i/2⌋ and ⌈i/2⌉ initial sub-polygons. For example, to find a convex merge option with six initial sub-polygons, combinations between convex merge options with three initial sub-polygons are only considered, instead of checking all possibilities (i.e., combinations between convex merge options with (1) one and five, (2) two and four, and (3) three initial sub-polygons). The details of the approximation are described as set in Algorithm 1.

**Algorithm 1:** Approximation for finding merge options
1:**function**FindingMergablePolygonOptions(*S*)2:    Ω←∅3:    $L_{1}\leftarrow S$4:    $L_{i}\leftarrow \emptyset$ for 2≤i≤|S|5:    **for** i=2 to |S| **do**6:        $n_{1}\leftarrow \left\lfloor i/2\right\rfloor$, $n_{2}\leftarrow \left\lceil i/2\right\rceil$7:        **for** j=1 to $|L_{n_{1}}|$ **do**8:           **for** k=1 to $|L_{n_{2}}|$ **do**9:               **if** Merged polygon of $S_{j}\in L_{n_{1}}$ and $S_{k}\in L_{n_{2}}$ is convex **then**10:                   Add $\left\{S_{j}\cup S_{k}\right\}$ to Ω and $L_{i}$11:               **end if**12:           **end for**13:        **end for**14:    **end for**15:    **return** Ω16:
**end function**



## 4. Analysis

### 4.1. Comparison with Existing Methods

**Figure 10:**
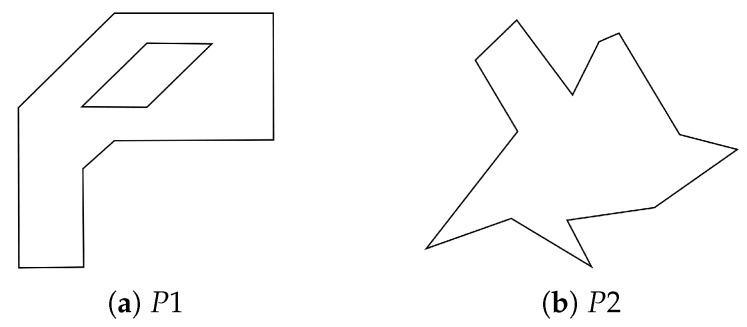
Illustrative examples for convex decomposition.

In this section, we investigate the proposed method by comparison with existing methods in the literature [8,11,14]. The method, implemented in the R programming language, is available at https://github.com/LasseDamtoft/InteriorEdgeExtension. Recall that [11] proposed a convex decomposition method with multiple sweep lines based on dynamic programming, and [8,14] proposed heuristics based on a greedy algorithm and the sweep line method, respectively. For comparison, we use illustrative polygons, P1 and P2, provided in the literature. The polygons are presented in Figure 10.

**Figure 11:**
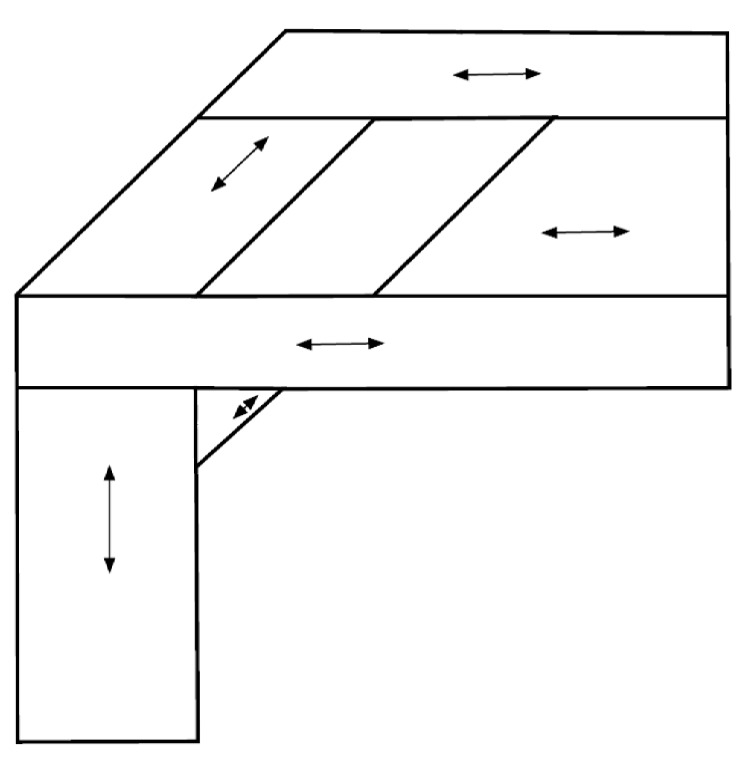
Convex decomposition for the polygon P1 by our method.

Our decomposition solution for the polygon P1 is presented in Figure 11. The arrows in the figure show the direction to cover the sub-polygons decomposed. We compared this solution with the solutions by the methods in [8,11] and the result is summarized in Table 1. Please refer to [8,11] for graphical illustrations of the benchmark solutions, respectively.

In the table, the relative gap to the best solution among the three solutions in terms of the total widths of the sub-polygons is presented. As can be observed, our method could provide a solution as good as the one by the method of [11], which searched most of the possible decomposition solutions. Note that our solution reached the solution with far fewer initial sub-polygons than the method of [11], because the method in [11] applied the sweep line algorithm to all edges of a polygon of interest, whereas we only extend certain edges at specific points. Compared with the sweep line method in [8], our method provided better performance. While having multiple decomposition directions seems beneficial for P1 as presented in Figure 11, the sweep line method in [8] could only split the polygon in a single direction (horizontal, in this example).

**Figure 12:**
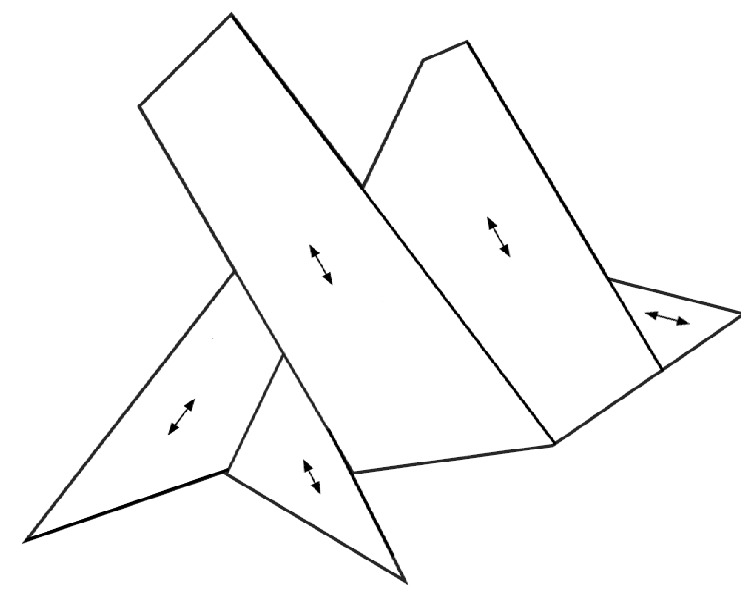
Convex decomposition for the polygon P2 by our method.

Next, we investigate our method, based on its result on the polygon P2. The convex decomposition for this polygon by our method is presented in Figure 12. The performance comparison with available solutions for the polygon in the literature is presented in Table 2. Please refer to [8,14] for graphical illustrations of the benchmark solutions, respectively. It should be noted that the solutions in the literature contained non-convex sub-polygons as, after deriving a convex decomposition, they applied a post-merge process if a certain condition on two adjacent convex sub-polygons was satisfied. Thus, the solution of our method with an arbitrary post-merge was also considered in the comparison.

In the comparison with P2, the sweep line method in [8] showed the best performance. The decomposition direction found by the sweep line method seemed to be optimal for minimizing the total width of the sub-polygons decomposed. Our method applied a different direction to decompose the left part of the P2. However, the performance gap was marginal. Our method also outperformed the heuristic in [14], which resulted in more sub-polygons than the other solutions.

Based on comparison with the existing convex decomposition methods presented, it can be concluded that our method provides competitive performance, in terms of its capability to minimize the total width of the sub-polygons and the computational complexity to find a solution.

### 4.2. Performance for a Complicated Polygon

We further investigate the performance of the proposed method with a complicated polygon, as the previous polygons were rather simple to decompose. We apply our method to a complicated polygon representing a layout of a port in Antwerp, Belgium. The CPP for an autonomous surface vehicle is often carried out to survey the seabed of a port, where the depth is critical for accommodating large-sized ships. Importantly, CPP is essential for most autonomous vehicles, ranging from ground vehicles (e.g., a robotic vacuum cleaner or a lawn mower), to aerial vehicles (an UAV), to over-/under-water vehicles (autonomous surface/underwater vehicles). As the complexity of the target area to cover varies depending on the operation scenario of the autonomous vehicles, the capability of handing a complicated polygon is key to wide application of the proposed method to various autonomous vehicles.

**Figure 13:**
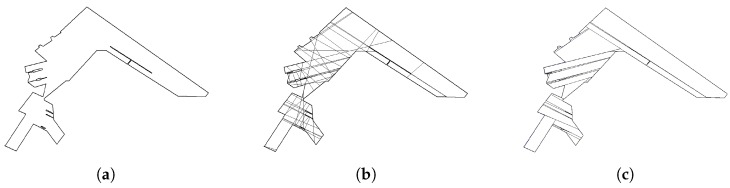
Output of the proposed method at each step to the polygon for Antwerp port layout: (**a**) Antwerp port layout; (**b**) initial sub-polygons after edge extension; and (**c**) the final convex decomposition solution.

The port layout is presented in Figure 13a. Given the layout, we first obtained initial convex sub-polygons by interior edge extension, as presented in Figure 13b. This step took 12 s, resulting in 238 initial sub-polygons. The convex merge option set Ω was, then, found in around 3 min with the approximation scheme in Algorithm 1, and the IP model (1)–(3) derived the final solution within three seconds. The final solution is presented in Figure 13c. From our test, the approximation scheme had a 4.3% performance gap, compared to the solution found without the scheme, while dramatically saving computation time.

## 5. Convex Decomposition under Multi-Unit Operational Environments

As the size of an area of interest becomes large, a single vehicle may not completely cover the area, necessitating the deployment of multiple units into the area. In this case, deriving coverage paths for multi-units in a conflict-free manner is crucial for guaranteeing the safety of the units and, in turn, a high service level of the units. In particular, for multi-UAV deployments, where the navigation and control of the units is more difficult than for other autonomous vehicles (e.g., automated guided vehicles), CPP becomes much more important [16,17].

A critical step toward the challenge is achieving an intelligent segmentation of an area of interest which can be covered by multiple vehicles; this segmentation is somewhat achieved by convex decomposition. Based on existing studies on service area segmentation [18], the convex decomposition method in this paper deserves further extension, with a focus on how critical the segmentation is, in terms of the quality of service at both fleet and individual vehicle levels.

For example, the proposed method in this paper can be embedded into an algorithm for multi-unit CPP. More specifically, the edge extension, the first step of the proposed method, can decompose a given area of interest into multiple sub-polygons. A vehicle routing problem is then solved to allocate the sub-polygons to multiple units and to derive a sequence of the allocated sub-polygons for each unit. The conflict between the sequences of sub-polygons, energy consumption of the units, and workload balance between the units are candidate constraints for the routing problem. Finally, following the sequences of the sub-polygons for each unit, the final coverage paths can be generated.

Note that the vehicle routing problem, a well-known NP-hard problem, is difficult to solve as the size of a problem instance (e.g., the number of units and sub-polygons) increases. To lessen the complexity of the problem, the IP model (1)–(3) can also be reformulated as a sub-polygon assignment model for multiple units. The solution of the updated IP model is, then, used as input to the TSP for each unit, which determines a sequence of sub-polygons for a final coverage path. An example of the reformulated IP model is written as below:

(4) $$\begin{array}{cc} \; & \min\;\sum_{j\in \Omega }\sum_{k\in K}w_{j}\cdot \lambda_{j}^{k}, \end{array}$$

(5) $$\begin{array}{ccc} s.t.\; & \sum_{j\in \Omega }\sum_{k\in K}a_{ij}\cdot \lambda_{j}^{k}=1 & \forall i\in S, \end{array}$$

(6) $$\begin{array}{ccc} \; & \sum_{k\in K}\lambda_{j}^{k}\leq 1 & \forall j\in \Omega , \end{array}$$

(7) $$\begin{array}{ccc} \; & \sum_{j\in \Omega }w_{j}\cdot \lambda_{j}^{k}-\sum_{j\in \Omega }w_{j}\cdot \lambda_{j}^{u}\leq \theta & \forall k,u\in K, \end{array}$$

(8) $$\begin{array}{ccc} \; & \lambda_{j}^{k}\in \left\{0,1\right\} & \forall j\in \Omega ,k\in K,, \end{array}$$

where the set *K* is a set of vehicles indexed by *k*, and $\lambda_{j}^{k}$ is an integer variable, which equals to one if merge option *j* is allocated to vehicle *k*, and zero otherwise. The objective function (4) minimizes the total width of the merge options allocated to vehicles. The constraint (5) ensures that all initial sub-polygons generated by the edge extension are assigned to one of the vehicles exactly once. The constraint (6) restricts merge option allocation to multiple vehicles. The constraint (7) guarantees that the workload difference between two vehicles cannot exceed a certain threshold, θ, for workload balancing. The constraint (8) is an integrality constraint on the decision variable.

## 6. Conclusions

This paper proposes a new convex decomposition method, which is essential for deriving a coverage path for an autonomous unit. Through comparison with existing convex decomposition methods, which have drawbacks in terms of their complexity and optimality, we demonstrate that the proposed method provides superior performance, with respect to the total width of the sub-polygons decomposed and the solution space searched by the method.

Considering the recent increasing demands in surveillance and area monitoring, CPP has become more significant and, so, the proposed convex decomposition method has a great number of potential applications, such as battlefield surveillance, maritime pollution monitoring, and target search (to name a few). We believe that the proposed convex decomposition method can play a key role in deriving high quality coverage paths and, accordingly, the success of such operations.

A further aspect of interest is in the use of the proposed method for real-world applications; in particular, using UAVs. While the proposed method in this paper is not unique in terms of supporting UAVs, as any automated or even manned solutions can utilize the results, the strengths of the method are highlighted in the areas where an autonomous vehicle provides significant advantages over manned solutions (e.g., in aerial surveillance and monitoring). Furthermore, there has been much on-going research into real-world applications of UAVs in surveillance and monitoring operations [19,20].

Note that, in a surveillance and monitoring operation using UAVs, the UAVs often deviate from their coverage paths, due to disturbances from their operational environments (e.g., wind) and the geometry of an area to cover can even change suddenly or over time, due to detected anomalies. These facts indicate the necessity for the ability to rapidly update the coverage path and, therefore, the computational complexity of the proposed method can be further investigated and improved, so as to provide a solution within a very short time.

From a practical perspective, the output of the method is directly implementable in any flight execution system using a geographic co-ordinate system. Thus, any basic UAV capable to fly-by-waypoint will be able to utilize the output from the method as input for coverage missions, with minor modifications to compensate for altitude and take-off/landing points.

## Figures and Tables

**Table 1:** Performance comparison with P1.

	**Multi-Sweep Line ([11])**	**Sweep Line ([8])**	**Ours**
Gap †	0.00	3.69	0.00

†: 100 (%)$\times \left(obj.value_{method}-obj.value_{best}\right)/obj.value_{best}$.

**Table 2:** Performance comparison with P2.

	**Greedy ([14])**	**Sweep Line ([8])**	**Ours**	**Ours with the Post-Merge**
Gap †	3.60	0.00	7.04	0.99

†: 100 (%)$\times \left(obj.value_{method}-obj.value_{best}\right)/obj.value_{best}$.
